# Longitudinal associations between perceived father and grandparent support of mothers in early childhood, and maternal mental health

**DOI:** 10.3389/fpsyt.2025.1507991

**Published:** 2025-06-02

**Authors:** Xinru Zhou, Chang Sun, Zhengjie Cai, Linhua Li, Yuju Wu, Hanwen Zhang, Hein Raat, Alexis Medina, Huan Zhou

**Affiliations:** ^1^ Department of Health Behavior and Social Medicine, West China School of Public Health and West China Fourth Hospital, Sichuan University, Chengdu, China; ^2^ Department of Gynecology and Obstetrics, Women’s Hospital, School of Medicine, Zhejiang University, Hangzhou, China; ^3^ Stanford Center on China’s Economy and Institutions, Freeman Spogli Institute for International Studies, Stanford University, Stanford, CA, United States; ^4^ Department of Public Health, Erasmus University Medical Centre, Rotterdam, Netherlands; ^5^ Stanford Center on China’s Economy and Institutions, Stanford University, Stanford, CA, United States

**Keywords:** maternal mental health issues, infant care assistance patterns, infant care transitions, rural China, longitudinal study

## Abstract

**Background:**

Maternal mental health issues have emerged as a public health concern garnering increasing attention in recent years, especially in low and middle-income countries (LMICs). The involvement of fathers and grandparents in infant care has been recognized as a key factor affecting maternal mental health. However, few studies have focused on the relationship between infant care assistance patterns and maternal mental health issues. This study aims to examine how infant care assistance patterns and its transitions over time affect maternal mental health in rural China.

**Method:**

This longitudinal study examined pregnant women in four rural Sichuan counties, China, using multi-stage random sampling to select 1054 mother-child dyads who completed baseline and follow-up assessments. We gathered four blocks of data: demographic characteristics, infant care assistance patterns (including sole maternal caregiving, joint parental caregiving, and maternal and grandparental caregiving), infant care assistance pattern transitions among mothers (including consistent assisted care, transitioned from sole mother care to assisted, transitioned from assisted care to sole mother, and sole mother care throughout) and maternal mental health issues (including depression, anxiety, and stress). Unordered multinomial logistic regression based on longitudinal data was used to explore the relationship between maternal mental health transitions and postnatal infant care assistance patterns transitions.

**Results:**

Of the mothers, 9.61% reported having severe depressive tendencies, 10.06% had moderate to severe anxious tendencies, and 4.65% reported having moderate to severe stress tendencies. Unordered multinomial logistic regression revealed that compared to mothers with consistent assisted care, those who transitioned from assisted care to sole mother care during the first-year postpartum experienced deterioration or a lack of improvement in depression (RRR=1.83, p<0.05), anxiety (RRR=2.07, p<0.05), and stress (RRR=2.37, p<0.01). Besides, mothers without assisted care throughout were also at higher risk of deteriorating or not improving in anxiety (RRR=1.86, p<0.05) and stress (RRR=2.36, p<0.05).

**Conclusion:**

This study shows a link between infant care assistance patterns and maternal mental health. Mothers transitioning from assisted to sole care in the first postpartum year may face declining or stagnant psychological health, suggesting that family members offer support in infant care to share the caregiving responsibilities.

## 1 Introduction

The perinatal period, a phase that includes pregnancy and the first year postpartum, is characterized by an elevated susceptibility to anxiety and depression for mothers (1). As many as one in five women experience perinatal depression or anxiety worldwide, with the proportion exceeding one in three in low- and middle-income countries (LMICs). (2, 3). In the rural areas of China specifically, a study found a much higher prevalence of perinatal mental health problems than the global average reported by the World Health Organization (4). A meta-study conducted in China reported a maternal depression prevalence of 16.3%, which is approximately 1.5 to 2 times higher than the prevalence rates observed in the general population (5). The situation is even more concerning in rural areas, where the prevalence rates of prenatal and postnatal depression were found to be 19.5% and 18.6%, respectively (6).

Perinatal mental health issues are not only associated with future depressive episodes, but they also pose risks to the mother-infant bonding process by impeding women from delivering the optimal care and stimulation that supports the child’s cognitive, psychosocial, and motor development (7, 8). Hence, maternal mental health issues have emerged as a public health concern garnering increasing attention in recent years.

Sources of maternal psychological vulnerability in the perinatal period include physiological factors, such as fluctuations in hormone levels (9), and parenting itself, which can cause anxiety, stress, and depression (10). Ideally, adverse maternal mental well-being gradually alleviates within the first year postpartum as hormone levels stabilize and mothers adapt to their role (11–13).

Unfortunately, sometimes maternal mental health issues persist after the first year (14). One of the often-overlooked causes for this is inadequate support from other family members (15, 16). If mothers are not provided with sufficient support from family members, they are more likely to have a worse mental health condition, such as postpartum depression (PPD) (16, 17).

The involvement of fathers and grandparents in infant care and their influence on maternal mental well-being has emerged as a critical area of research (18, 19). Generally, there are three types of family infant care assistance patterns in LMICs: sole mother caregiving, where the mother cares for the infants alone; joint parental caregiving, where the father assists the mother in caring for the infants; and maternal and grandparental collaborative caregiving, where one or more grandparents assist the mother (20). Studies pointed out that paternal involvement in infant care could provide mothers with multidimensional everyday support (including financial, practical, and emotional support), which can promote maternal mental health (21). And growing body of evidence has revealed that a lack of parenting support from fathers may increase the risk of maternal psychological distress (21–26). Mothers who feel supported by their partners have been found to show better mental health during the first five years after birth (27). While paternal involvement has shown promising protective effects against maternal distress, studies examining the relationship between grandparent involvement in infant care and maternal mental well-being found mixed results (1, 13, 28, 29). Research indicates that grandparental involvement in childcare may help in the adaptive transition to motherhood by alleviating the mother’s burden (1). However, other studies have pointed out that conflicts or interference from grandparents can have negative effects on mothers’ emotional state and parenting practices (1, 12, 13). Leung and Lam have reported that conflict between new mothers and grandparents contributes to postpartum depression, particularly among Chinese (13). The impact of grandparental involvement thus appears multifaceted and requires further investigation.

Furthermore, infant care assistance patterns are not fixed and can change due to changes in family structure (30). Lee and Hung (17) have noted the dynamic nature of support during the postpartum period. They pointed out that caregiving individuals may experience support growth, support decay, and support stativity, each trajectory carries different implications for maternal mental health (17). Some studies have focused on examining the impact of infant care assistance patterns involving the involvement of fathers or grandparents on maternal mental health, but few of them explored the impact of transitions in infant care assistance patterns on maternal psychological well-being.

Mothers in rural China are especially at risk of maternal mental health issues due to limited access to resources and the often-overlooked frequent transitions in their care assistance patterns. Compared to mothers in urban China, rural mothers frequently face higher levels of stress due to economic hardship, limited access to healthcare, and the pressures of traditional gender roles, which expect them to balance child-rearing, household chores, and agricultural work (31, 32). Moreover, rural Chinese families typically go through much more transitions in infant care assistance patterns as family members, usually fathers, leave home for work in cities (33). According to the National Bureau of Statistics of China, by the end of 2017, there were 28.65 million farmer migrant workers, with 13.71 million (79.78%) employed in cities outside their townships (34). Hence, postpartum mothers in rural regions face increased disruptions in family support. However, previous research on maternal perinatal mental health mainly focused on influences of partner support or grandparental support and neglected the influence of constant changes in support from family members on maternal mental health—a relationship that has been rarely examined empirically.

To sum up, studies on how infant care assistance patterns affects maternal psychological well-being have mixed results, and few previous studies explored the impact of transitions in infant care assistance patterns. Therefore, this study aims to examine the relationships between infant care assistance patterns, their transitions over the first year postpartum, and maternal mental health issues in rural China. To meet this goal, we pursued three specific objectives. First, we described the status of family caregiving patterns and maternal psychological well-being in rural western China. Second, we identified correlations between infant care assistance patterns and maternal mental well-being. Third, we examined the longitudinal relationship between transitions in infant care assistance patterns and postpartum changes in maternal mental well-being based on longitudinal data.

## 2 Methods

### 2.1 Study design and participants

This study uses data from a longitudinal study of mothers conducted in four rural counties within Nanchong Municipality of Sichuan Province, China. We used a multi-stage cluster sampling method to select the sample. Participants were selected based on a list of all pregnant women in the selected townships provided by the local perinatal healthcare system. All women who were at least 14 weeks into gestation as well as mothers with infants younger than six months enrolled in the study. Baseline data were collected between July and August of 2021, and follow-up data were collected one year later in 2022. Trained enumerators conducted computer-assisted home visit interviews to collect information from all participants using a structured and pre-tested questionnaire.

A total of 1,054 mother-child pairs, with all mothers serving as the primary caregivers, completed both baseline and follow-up assessments and were included in the final analysis. All participants provided their informed consent prior to enrollment and understood that their participation was entirely voluntary. This study was approved by the Sichuan University Medical Ethics Committee (K2019046) (Chengdu, China), Stanford University (44312) (Stanford, CA, USA), and the University of Nevada, Reno (1737966-1) (Reno, NV, USA).

### 2.2 Care pattern assessment

#### 2.2.1 Definitions and measurements of infant care assistance patterns

**Figure 1:**
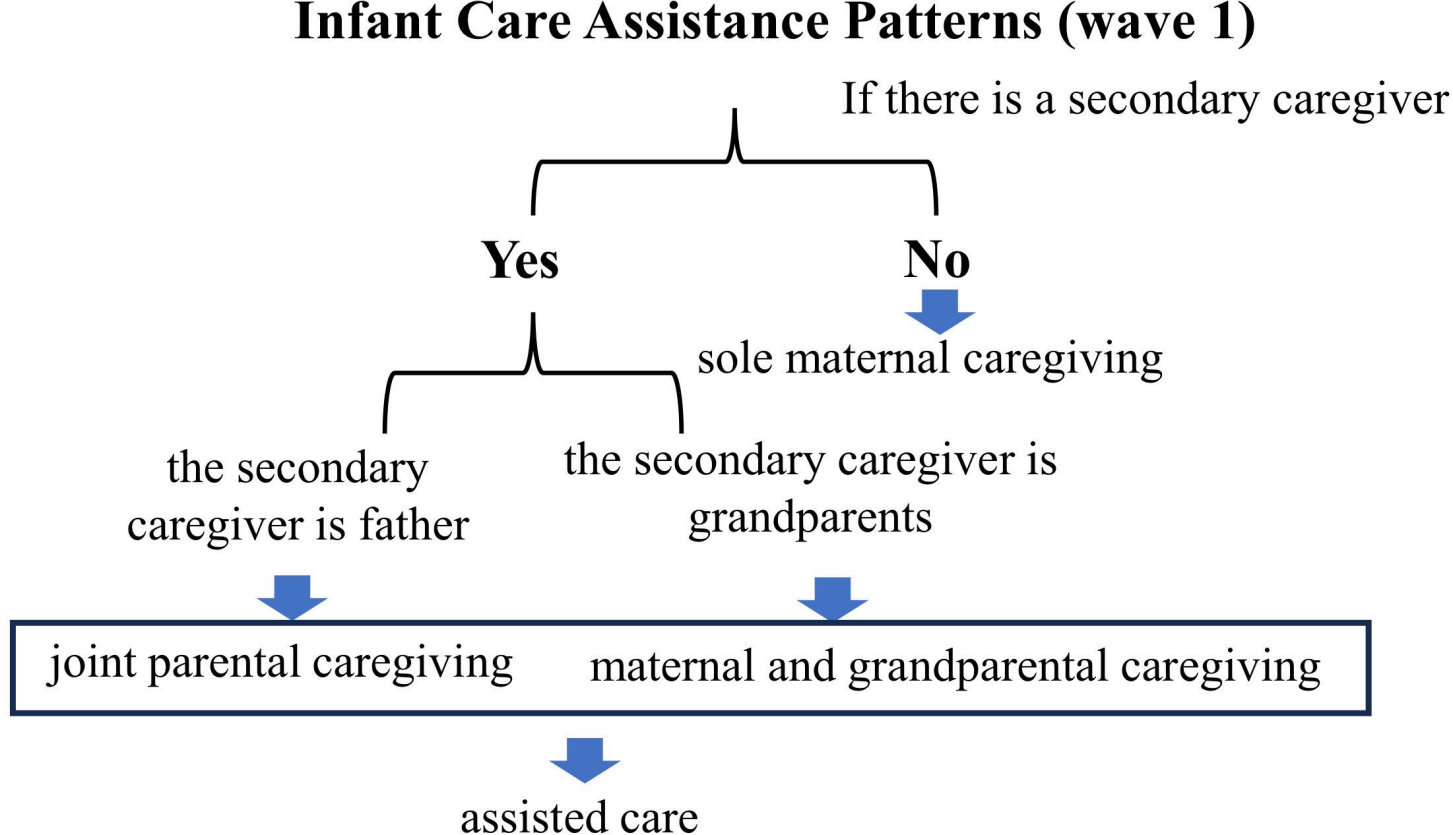
Infant care assistance patterns (wave 1).

During the follow-up assessment conducted postpartum for all participating mothers, we systematically gathered information concerning the presence and roles of secondary caregivers for infants and toddlers, with the mother assuming the primary caregiving role. This assessment aimed to document the various patterns of infant care assistance, including sole maternal caregiving, joint parental caregiving, and maternal and grandparental caregiving (See **Figure 1**).

Sole maternal caregiving is defined as a scenario where the mother alone continuously cares for the infant. Joint parental caregiving is defined as a situation where the father assists the mother in caring for the infant. To measure it, interviewers asked mothers whether there is a secondary caregiver present, and identifying if this caregiver is the father. Maternal and grandparental caregiving is defined as a situation where one or more grandparents assist the mother (Note: 98.6% of the “grandparents” category were grandmothers, with grandfathers accounting for 1.4% of cases; given this imbalance, we did not disaggregate by grandparent gender). Similar to joint parental caregiving, the presence and identity of any secondary caregivers (in this case, grandparents) are confirmed during the assessment. We operationally defined joint parental caregiving and maternal and grandparental caregiving as assisted care.

#### 2.2.2 Postnatal infant care assistance patterns transitions

**Figure 2:**
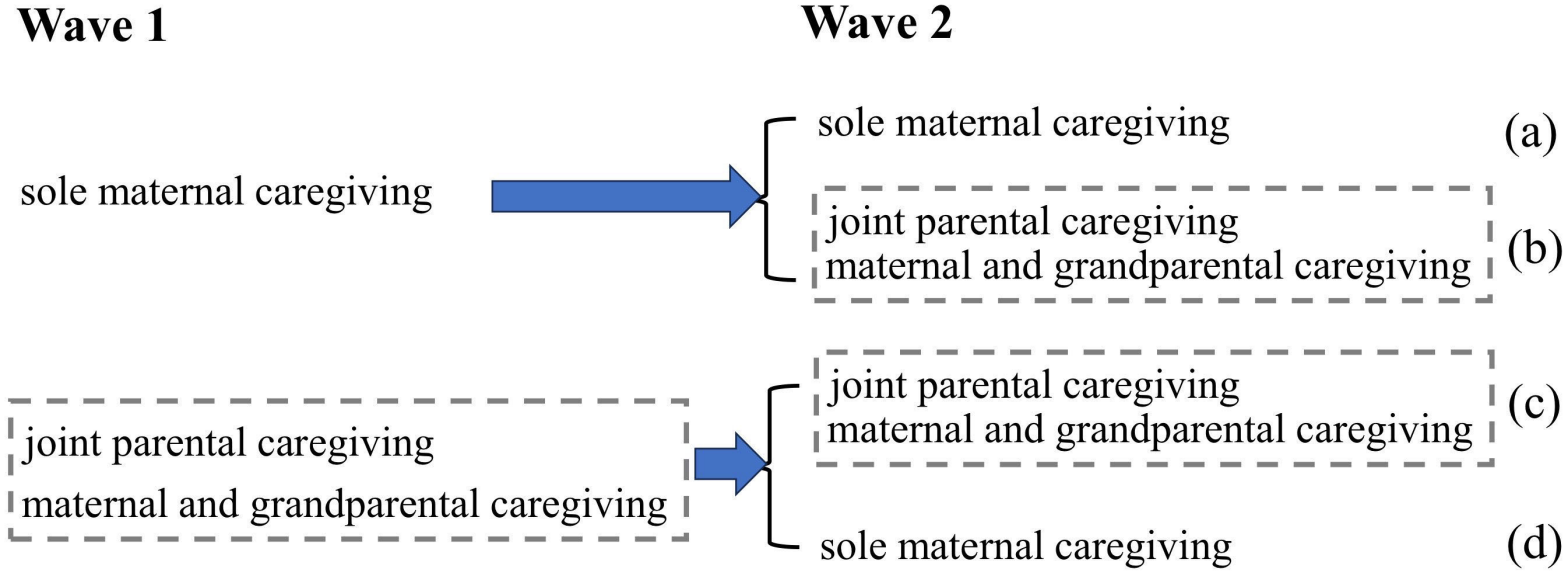
Infant care assistance patterns transitions (two waves). **(a)** sole mother care throughout; **(b)** transitioned from sole mother care to assisted; **(c)** consistent assisted care; **(d)** transitioned from assisted care to sole mother.

During the follow-up assessment, we collected data on the postnatal infant care assistance patterns transitions among mothers. These transitions were categorized based on changes in the presence of secondary caregivers assisting the mother in caring for the infant or toddler postpartum. The categories included: consistent assisted care (continued assistance from a secondary caregiver postpartum), transition from sole mother care to assisted (a transition from solely maternal care to receiving assistance from a secondary caregiver), transition from assisted care to sole mother care (a transition from receiving assistance from a secondary caregiver to solely maternal care), and sole mother care throughout (sole maternal caregiving postpartum) (See **Figure 2**).

### 2.3 Outcome measures

Maternal mental health is measured at both baseline and follow-up evaluations in three dimensions: depression, anxiety, and stress. Maternal Depression was assessed using the Edinburgh Postnatal Depression Scale (Cronbach’s α = 0.83). Scores were categorized into three groups: no depressive tendencies (Edinburgh Postnatal Depression Scale score < 11), mild depressive tendencies (score11-12), and severe depressive tendencies (score ≥ 13) (35). Maternal anxiety and maternal stress were measured using the Depression Anxiety and Stress Scale (DASS-21). In our study, the DASS-21 anxiety subscale had Cronbach’s α = 0.73, the stress subscale α = 0.84, and the overall DASS-21 α = 0.92. Maternal Anxiety was classified into three groups: no anxious tendencies (DASS-21 Scale anxiety dimension score ≤ 7), mild anxious tendencies (scores 8-9), and moderate to severe anxious tendencies (score ≥ 10) (36). Maternal stress was categorized as no stress tendencies (DASS scale stress dimension score ≤ 14), mild stress tendencies (scores 15-18), and moderate to severe stress tendencies (score ≥ 19) (36). For both maternal anxiety and stress, scores classified as moderate and severe were merged into one group because each category made up less than 5% of the total sample.

Based on the two waves of assessments, we categorized the changes in maternal mental health into three distinct trajectories: the absence of mental health issues throughout (no mental health issues = scoring “no tendencies” in all three dimensions in both baseline and follow-up surveys), improvement in mental health (mental health issue present at baseline but improved or normalized in the follow-up), and deterioration or no improvement (mental health issue present at baseline and worsened or showed no improvement in the follow-up).

### 2.4 Covariates

Various confounders such as maternal age at delivery, maternal education (junior high school or less lower vs. high school vs. college or university), parity (primipara vs. multipara), infant sex (male vs. female), infant age at follow-up (in months), maternal perceived social support (in scores), annual per capita income of the family (in CNY), and household assets (whether the household owned or had access to a water heater, washing machine, refrigerator, air conditioner, television, computer, motorcycle, and car or truck) were assessed in the baseline or follow-up survey. We calculated participants’ socioeconomic status (SES) based on maternal education, the annual per capita income of the family, and household assets. A principal component analysis (PCA) was performed to obtain each participant’s SES score, which was then sorted into one of four categories: low level (Q1), relatively low level (Q2), relatively high level (Q3), and high level (Q4).

### 2.5 Statistical analyses

To examine the association between maternal psychological well-being and infant care assistance patterns, we employed ordered multivariate logistic regressions with robust standard errors. These analyses were adjusted for covariates, the corresponding outcomes at baseline, and subcounty fixed effects. The estimation of adjusted β coefficients and corresponding 95% confidence intervals was conducted.

To explore the relationship between maternal psychological well-being transitions and postnatal infant care assistance patterns transitions, we employed unordered multinomial logistic regression with robust standard errors. These analyses were adjusted for covariates and subcounty fixed effects. The estimated relative risk ratio (RRR) and corresponding 95% confidence intervals were recorded.

All statistical analyses were performed using STATA 16.0 with a two-sided significance level of p = 0.05.

## 3 Results

### 3.1 Sample characteristics

We collected comprehensive data on 1,054 mother-child pairs. **Table 1** presents a summary of the characteristics of the mother-infant dyads at baseline and follow-up. At baseline, 52.75% of the infants had not yet been born. Among the mothers of infants who had already been born, 48.29% were under 28 years old, and the majority were multiparous (71.25%). Most mothers (63.38%) reported having an education level of junior school or lower. The median annual per capita income was 12,400 RMB. Based on socioeconomic status (SES), 27.89% of mothers were classified as having low SES. Among the 47.25% of infants born at baseline, 52.81% were male. The median score of maternal perceived social support at baseline was 5.42. At follow-up, the infant age distribution ranged from 0 to 18 months, with 47.82% aged 6–11 months, and 50.09% of infants were male. The median score of maternal perceived social support at follow-up was 5.33.

**Table 1:** Descriptive characteristics of mother and infant pairs included in the analysis (N = 1054).

Characteristics	Baseline wave	Follow-up wave
Maternal age (years)		
∼28	509 (48.29)	–
29-34	406 (38.52)	–
35∼	139 (13.19)	–
Maternal education level		
Junior school or lower	668 (63.38)	–
Senior school	247 (23.43)	–
College or university	139 (13.19)	–
Annual per capita income^*^	1.24 (0.80 - 2.00)	–
SES		
Low	294 (27.89)	–
Relatively lower	265 (25.14)	–
Relatively higher	259 (24.57)	–
High	236 (22.39)	–
Parity		
Multipara	751 (71.25)	–
Primipara	303 (28.75)	–
Infant sex		
Male	263 (52.81)	528 (50.09)
Female	235 (47.19)	526 (49.91)
Infant month age		
Unborn	556 (52.75)	0
0–5 months	498 (47.25)	234 (22.20)
6–11 months	0	504 (47.82)
12–18 months	0	316 (29.98)
Maternal perceived social support^*^	5.42 (4.58-6.00)	5.33 (4.50-6.00)
Maternal depression		
No depressive tendencies	845 (80.17)	914 (86.72)
Mild depressive tendencies	130 (12.33)	66 (6.26)
Severe depressive tendencies	79 (7.50)	74 (7.02)
Maternal anxiety		
No anxious tendencies	845 (80.17)	904 (85.77)
Mild anxious tendencies	65 (6.17)	44 (4.17)
Moderate to severe anxious tendencies	144 (13.66)	106 (10.06)
Maternal stress		
No stress tendencies	922 (87.48)	948 (89.94)
Mild stress tendencies	67 (6.36)	57 (5.41)
Moderate to severe stress tendencies	65 (6.17)	49 (4.65)
Infant care assistance patterns (Secondary caregiver)		
Sole maternal caregiving (None)	274 (26.00)	426 (40.42)
Joint parental caregiving (Father)	145 (13.76)	106 (10.06)
Maternal and grandparental caregiving (Grandparents)	635 (60.25)	522 (49.53)

*M (Q_1_, Q_3_).

In terms of maternal mental health, 80.17% of mothers at baseline showed no depressive tendencies, 12.33% had mild depressive tendencies, and 7.50% exhibited severe depressive tendencies; at follow-up, the proportion of mothers without depressive tendencies increased to 86.72%. Regarding anxiety and stress, 80.17% of mothers at baseline reported no anxiety, and 87.48% experienced no stress, with these proportions rising at follow-up to 85.77% and 89.94%, respectively. Infant care assistance patterns showed that at baseline, 60.25% of caregiving involved both maternal and grandparental caregiving, while by follow-up, sole maternal caregiving increased to 40.42%.

### 3.2 Association between maternal psychological well-being and infant care assistance patterns

**Table 2** presents the findings of the multivariate regression model examining the associations between maternal mental health and infant care assistance patterns. We found statistically significant negative associations between maternal and grandparental caregiving and maternal depression (β=-0.57, 95% CI: -1.136, -0.013), maternal anxiety (β=-0.72, 95% CI: -1.226, -0.215), and maternal stress (β=-1.16, 95% CI: -1.819, -0.504). In other words, mothers who received care assistance from the infant’s grandparents were more likely to exhibit lower levels of maternal psychological distress (depression, anxiety, and stress) than mothers who cared for their infants alone.

**Table 2:** Association between maternal mental health and infant care assistance patterns among infants aged 0-18 months in rural western China (N=1054).

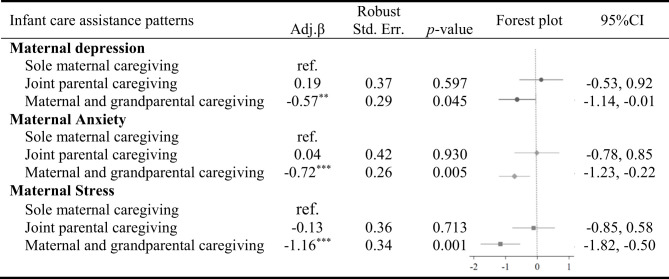

Adjusted coefficient controlled for the following covariates: the household socioeconomic status (derived from maternal and paternal educational levels, annual per capita income, and household ownership assets), maternal perceived social support, maternal age, infant age, parity, the corresponding outcomes at baseline, and subcounty fixed effects. Robust standard errors are clustered at the village level. * p<0.05, ** p<0.01, *** p<0.001.

### 3.3 Relationship between maternal psychological well-being transitions and postnatal infant care assistance patterns transitions

**Table 3** presents the findings of the multivariate regression model examining the relationship between maternal mental health transitions and postnatal infant care assistance patterns transitions in the first year postpartum. Compared to mothers with consistent assisted care, those who transitioned from assisted care to sole mother care experienced a deterioration or lack of improvement in depression (RRR=1.83, p<0.05), anxiety (RRR=2.07, p<0.05), and stress (RRR=2.37, p<0.01). Besides that, mothers who care for the infant alone throughout also had a higher risk of deteriorating or not improving in terms of anxiety (RRR=1.86, p<0.05) and stress (RRR=2.36, p<0.05).

**Table 3:** Relationship between maternal mental health transitions and postnatal infant care assistance patterns transitions within the first year postpartum in rural Western China (n=1054).

Infant care assistance pattern transitions	Maternal depression		Maternal anxiety		Maternal stress	
	Univariate RRR	Multivariate RRR	Univariate RRR	Multivariate RRR	Univariate RRR	Multivariate RRR
Mothers with improvement in the mental health state vs. Mothers without such issue persistently						
Consistent assisted care	ref.	ref.	ref.	ref.	ref.	ref.
Transitioned from sole mother care to assisted	0.45	0.44	0.52	0.57	1.09	1.26
	(0.24)	(0.23)	(0.29)	(0.33)	(0.53)	(0.65)
Transitioned from assisted care to sole mother	0.76	0.77	1.23	1.27	0.80	0.81
	(0.21)	(0.21)	(0.32)	(0.34)	(0.30)	(0.29)
Sole mother care throughout	1.23	1.19	1.18	1.34	0.98	1.16
	(0.31)	(0.30)	(0.31)	(0.38)	(0.32)	(0.43)
Mothers with deterioration or no improvement vs. Mothers without such issue persistently						
Consistent assisted care	ref.	ref.	ref.	ref.	ref.	ref.
Transitioned from sole mother care to assisted	1.12	1.04	0.86	0.79	0.83	0.81
	(0.53)	(0.52)	(0.42)	(0.40)	(0.54)	(0.53)
Transitioned from assisted care to sole mother	1.79*	1.83*	1.98*	2.07*	2.16*	2.37**
	(0.49)	(0.51)	(0.60)	(0.61)	(0.72)	(0.77)
Sole mother care throughout	1.65	1.46	1.94*	1.86*	2.24*	2.36*
	(0.45)	(0.49)	(0.59)	(0.58)	(0.77)	(0.86)
Control Variables	None	Yes	None	Yes	None	Yes

Control variables were prespecified and include the household socioeconomic status (derived from maternal and paternal educational levels, annual per capita income, and household ownership assets), maternal perceived social support, maternal age, infant age, parity, and subcounty fixed effects. Robust standard errors in parentheses are clustered at the village level. RRR, relative risk ratio. *p<0.05, **p<0.01.

## 4 Discussion

### 4.1 Main findings

Perinatal maternal mental health is a public health priority that has received significant global attention in recent years. Improving maternal psychological well-being is a focus of the Sustainable Development Goals (SDGs) and the Global Strategy for Women’s, Children’s, and Adolescents’ health (37). Better identification of modifiable risk factors for maternal psychological well-being would inform public health and clinical approaches to preventing these risk factors. In this study, we examined the static and dynamic aspects of the relationship between infant care assistance patterns and maternal psychological well-being in rural China.

Consistent with previous research, we found that mothers who received care assistance from the infant’s grandparents had lower levels of maternal mental health issues compared to those caring for their infants alone. However, assistance from the infant’s father did not lead to a statistically significant reduction in maternal mental health issues as maternal and grandparental caregiving did, which is somewhat inconsistent with most previous studies (21, 22).

Several factors might explain our results. First, recent studies have found that younger mothers often show lower resilience and have worse mental health outcomes (11, 15). Compared to fathers, grandparents often have more parenting experience and time resources which may make them more effective in providing practical support through direct involvement in infant care and emotional support (1, 18). Hence, grandparents are often the first people who mothers approach for help or advice with parenting (1). Grandparental involvement is important for mothers to learn infant care and adapt to their new role. Quickly and effectively adapting to the role of motherhood can lower the risk of psychological disorders (11).

Second, support is important for ensuring psychological stability during the perinatal period (1). In the first few months postpartum, mother without sufficient emotional and practical support from others are at risk for poor mental health (38). There is agreement among previous works that a greater level of support is associated with a lower incidence of maternal mental health disorders (18). However, in rural China, although fathers have been aware of the importance of their involvement to give support for mothers as key family members, economic pressures still necessitate a strong need for them to work away from home (39). Additionally, fathers’ involvement might not meet mothers’ expectations regarding the level or quality of assistance. And the relation between partner support and maternal mental health depends on the quality of paternal involvement (27). For example, within the cultural context of rural China, fathers are typically seen as the economic providers for the family, while the role of infant care is predominantly assumed by females (39). This traditional division of gender roles may limit fathers’ practical involvement in infant care, even if they are willing. Therefore, the demand for the involvement of grandparents in caregiving remains high. Assistance from grandparents in infant care provides mothers with not only practical support but also emotional support. Practical support means giving mothers the physical resources needed to cope with the demands of parenting, including directly assisting the parent in child care or housework. Emotional support, on the other hand, provides mothers confidence to undertake the parenting role (40). Additionally, assistance from grandparents in infant care allows mothers to venture outside of the home, form new social connections or strengthen existing ones, and thus receive social support (41).

Third, Prime et al. found that financial insecurities contributed to worse maternal mental health (2020). Assistance from grandparents enables mothers to return to work and increases family income, while support provided by grandparents also alleviates the financial burden of the family (42, 43).

More importantly, longitudinal data shows that mothers who transitioned from assisted care to sole mother care during the first-year postpartum experienced deterioration or lack of improvement in psychological well-being. And our findings uniquely highlight that the withdrawal of this support—when mothers transition to sole care—leads to a decline in maternal mental health, a dynamic less explored in previous research. Furthermore, interestingly, we found that mothers who transitioned from sole mother care to assisted care did not experience significant improvements in their psychological well-being, suggesting that the timing and continuity of care assistance might be critical factors.

Our results have implications for public health practice, and highlight the critical need for assisted care during this vulnerable period. We recommend that other family members engage in the caregiving of infants to provide practical and emotional support to the primary caregivers—typically the mothers. This involvement can substantially improve the maternal mental health. Of note, it is essential that such involvement is consistent, as initial support can be crucial for improving maternal mental health.

### 4.2 Strengths and limitations

This study has several strengths. First, it advances the limited body of literature on the impact of assisted care patterns on maternal psychological well-being, with a particular emphasis on the critical role of grandparents’ involvement. Second, the use of longitudinal data to explore the causal relationship between infant care patterns and maternal psychological well-being lends greater credibility to our findings. This study also has limitations. Although our statistical analysis took into account many confounding variables, there may still be confounding factors that we did not observe. Additionally, our data did not permit separate analyses of grandmother versus grandfather caregiving due to the low prevalence of grandfathers (< 5%). Future research should explicitly examine grandfathers’ roles and how their caregiving experiences and strategies may differ from those of mothers, fathers, and grandmothers. Furthermore, although our longitudinal component includes two waves, it does not permit continuous tracking of caregiving changes beyond 18 months. Future research should implement more frequent or extended follow-ups to explore how infant-care assistance patterns evolve over the long term as children grow.

## 5 Conclusion

This study examined the relationship between infant care assistance pattern, its transition over time, and maternal psychological well-being in rural China. Results showed that mothers transitioning from assisted care to sole mother care in the first year postpartum experienced a decline or stagnation in mental health, as measured by depression, anxiety, and stress. Further research is wanting to understand the effects of changing family support on maternal psychological well-being. Longitudinal studies that examine the roles of fathers and grandparents in infant care, along with detailed assessments of maternal mental health, can provide especially valuable insights for research and practice. Finally, as this study confirms that sustained assistance is associated with better maternal mental health in rural China, encouraging family members to offer sustained support to mothers in child-rearing could significantly reduce the risk of maternal mental health disorders in the region.

## Data Availability

The raw data supporting the conclusions of this article will be made available by the authors, without undue reservation.
